# *In vitro* validation of an ultra-sensitive scanning fluorescence microscope for analysis of Circulating Tumor Cells

**DOI:** 10.1111/apm.12183

**Published:** 2013-10-29

**Authors:** Thore Hillig, Ann-Britt Nygaard, Laura Nekiunaite, Jörg Klingelhöfer, György Sölétormos

**Affiliations:** 1 Department of Clinical Biochemistry, Hilleroed HospitalHilleroed; 2 Department of Neuroscience and Pharmacology, Faculty of Health Sciences, University of CopenhagenKbh N, Denmark

**Keywords:** Circulating tumor cells, CTC, cancer, CytoTrack

## Abstract

Analysis of circulating tumor cells (CTC) holds promise of providing liquid biopsies from patients with cancer. However, current methods include enrichment procedures. We present a method (CytoTrack®), where CTC from 7.5 mL of blood is stained, analyzed and counted by a scanning fluorescence microscope. The method was validated by breast cancer cells (MCF-7) spiked in blood from healthy donors. The number of cells spiked in each blood sample was exactly determined by cell sorter and performed in three series of three samples spiked with 10, 33 or 100 cells in addition with three control samples for each series. The recovery rate of 10, 33 and 100 tumor cells in a blood sample was 55%, 70% and 78%, percent coefficient of variation (CV%) for samples was 59%, 32% and 18%, respectively. None of the control samples contained CTC. In conclusion, the method has been validated to highly sensitively detect breast cancer cells in spiking experiments and should be tested on blood samples from breast cancer patients. The method could benefit from automation that could reduce the CV%, and further optimization of the procedure to increase the recovery.

Cancer is one of the most common causes of death in the western world (1). It does not generally become critical until it develops the ability to invade surrounding tissue and spread from the primary tumor to other parts of the body through the blood or lymphatic systems. Majority of all cancer deaths are attributed to the metastatic spread of cancer (2).

The existence of circulating tumor cells (CTC) – malignant cells of epithelial origin – has been known since 1869 (3) and has been related to metastasis (4). Escape of cancer cells to the circulation is an important step in the process leading to the formation of metastases (Fig. 1).

**Fig. 1:**
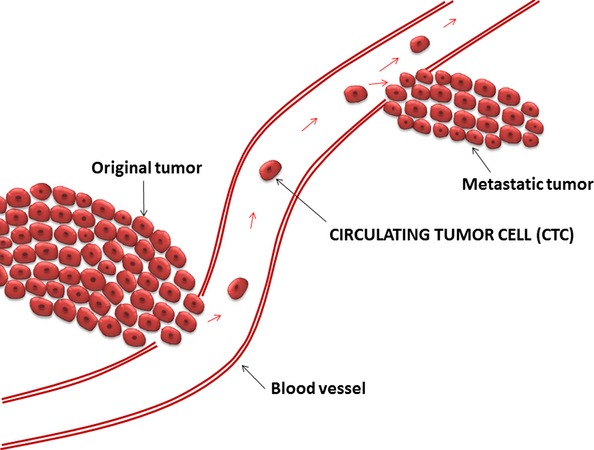
Illustration of circulating tumor cells (CTC) forming metastasis. Tumor cells escape from the primary tumor site into the circulation where they can be detected as CTC. Some cells will eventually extravasate from the circulation into the surrounding tissue and form metastasis at distant organs.

In principle, the presence of CTC is proof that a patient has cancer (5–7). This makes CTC a potential prognostic and predictive tumor marker in the diagnosis, treatment and monitoring of cancer. Several studies have shown CTC to be an early marker of tumor progression and hold independent prognostic information compared with scanning techniques (8,9). The accessibility of a peripheral blood sample enables sequential sampling during therapy and thus CTC could be used in line with other tumor biomarkers to monitor the efficiency of a treatment. The number of CTC is reduced if a treatment is effective, but if cancer cells lose sensitivity to the applied therapy, the number of CTC will remain unchanged or even increase (6). Thus, the number of CTC by itself is a valuable tumor biomarker.

The analysis of CTC holds promise as a method to improve the possibilities for personalized health care through molecular characterization of CTC (10,11). However, first, there remains the difficult task of actually finding the rare CTC. Many concepts have been suggested and technologies tested, including magnetic sorting, filtration based on size, dielectrophoresis, spinning columns, etc. (12–15). However, all of the above methodologies require enrichment procedure before staining, analysis and characterization. Enrichment involves positive or negative selection of certain cells and introduces an in-built bias to the CTC analysis. To avoid the enrichment procedure, we tested and validated a method for which all cells in a standard blood sample are stained, mounted on a glass disk and scanned with an automated fluorescence scanning microscope (Fig. 2). The purpose of this study was to determine the accuracy, precision and linearity of the scanning fluorescence microscope method.

**Fig. 2:**
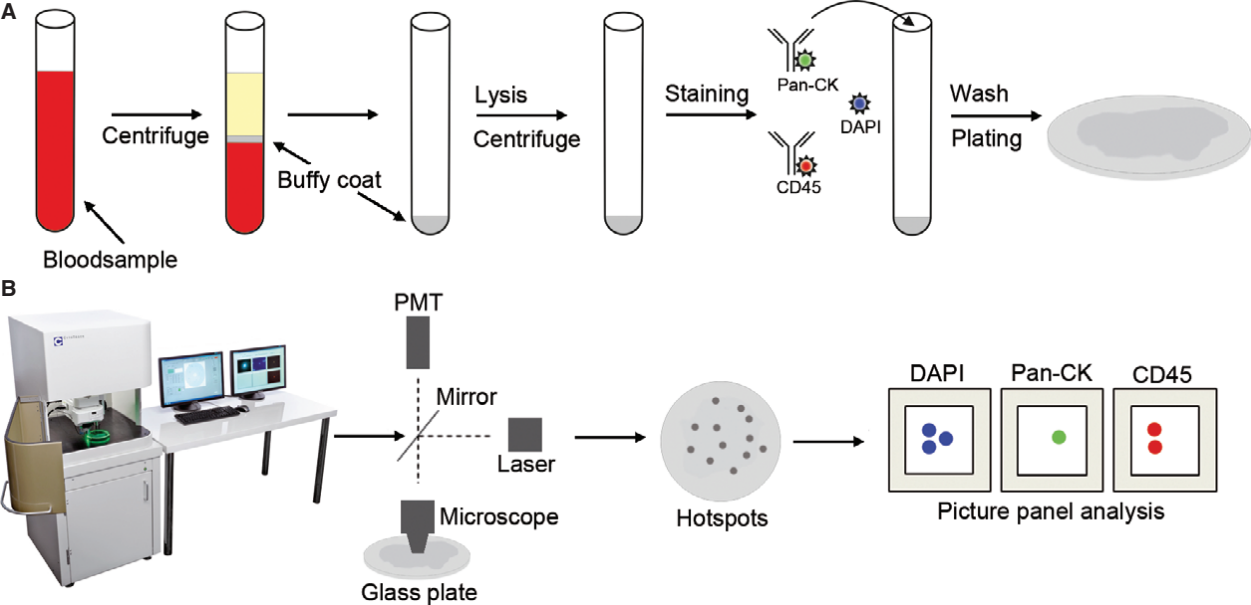
The workflow of detecting circulating tumor cells (CTC) in blood samples of cancer patients using the CytoTrack rare cell detection system. (A) Sample preparation of a blood sample for CTC analysis requires centrifugation, isolation of buffy coat, red blood cell lysis, staining, wash and final application of cells to scanning glass disk. (B) The scanning of a disk with the applied sample is performed in an outward spiral pattern. During this procedure, the stained cells are excited with a laser at 488 nm. The signals are detected by a photomultiplier tube (PMT). The positions on the disk with possible CTC are recorded as hotspots. The hotspots are selected for picture acquisition where pictures are taken in the 4,6-diamidino-2-phenylindole channel, the Alexa-Fluor488 channel and the Cy5 channel, and a picture panel is generated.

## Materials and Methods

### Cell culture

The human breast adenocarcinoma cell line MCF-7 was obtained from ATCC (American Type Culture Collection, Manassas, VA, USA). The MCF-7 cells were cultured in Dulbecco's Modified Eagle (DMEM)-GlutaMAX™ medium (Gibco, Grand Island, NY, USA), supplemented with 1% penicillin-streptomycin and 10% heat-inactivated fetal calf serum (FCS). The culture was grown in cell culture flasks in a humidified atmosphere containing 5% CO_2_ at 37 °C. Cell culture was washed with Dulbecco's phosphate-buffered saline without calcium and magnesium (DPBS, Gibco) followed by harvesting with TrypLe Express (Gibco). Cells were counted with a NucleoCounter® SP-100™ (ChemoMetec, Alleroed, Denmark).

### Flow cytometry

One million MCF-7 cells and 100 μL blood cells were fixed with 900 μL FACS Lysing Solution (BD Bioscience, San Jose, CA, USA) and washed with 1 mL DPBS with 1% bovine serum albumin (BSA, VWR, Glostrup, Denmark). Then cells were permeabilized with DAKO Intrastain permeabilization buffer (DAKO, Glostrup, Denmark) and stained with 4,6-diamidino-2-phenylindole (DAPI; VWR, Glostrup, Denmark), a Cy5-conjugated anti-CD45 antibody (HI30 eBioscience Inc., San Diego, CA, USA) and an Alexa Fluor 488-conjugated anti-pan-cytokeratin antibody (AE1/AE3 eBioscience Inc.). After staining, cells were washed with PBS with 1% BSA and resuspended in FACSFlow solution (BD Bioscience) and analyzed on a FACSCalibur flow cytometer (BD Biosciences). Negative controls using Alexa Fluor 488- or CY5-conjugated Mouse IgG1 isotype control (SouthernBiotech, New Orleans, LA, USA) were performed for evaluating the specificity of the antibodies. The results were analyzed with CellQuest Pro software (BD Biosciences).

### Blood sample collection

Peripheral blood (7.5 mL) was collected from a healthy donor into CellSave Preservative Tubes (Veridex LLC, Raritan, NJ, USA), which are evacuated blood draw tubes containing EDTA and a cellular preservative. Blood samples were maintained at room temperature for different time intervals and processed within 48 h.

### Sample processing

Samples were centrifuged at 2500 *g* for 10 min and the layer with mononuclear cells including tumor cells were transferred to a 15-mL tube. Red blood cells were lysed with FACS Lysing solution (BD Biosciences) and the samples centrifuged at 2500 *g* for 10 min. Thereafter, cells were permeabilized with DAKO Intrastain permeabilization buffer (DAKO) and stained for 30 min in dark at 4 °C with following reagents: a Cy5-conjugated CD45 antibody (HI30 eBioscience Inc.), and an Alexa Fluor 488-conjugated pancytokeratin antibody (AE1/AE3 eBioscience Inc.). Cells were then washed three times with PBS with 1% BSA and resuspended in H_2_O, transferred to a glass disk, with a radius of 60 mm, air-dried, and mounted using Vectashield Hard Set mounting medium with DAPI H-1500 (Vector Laboratories Inc., Burlingame, CA, USA; Fig. 2A).

### Scanning

The glass disk with stained and mounted cells was counted and analyzed by a scanning fluorescence microscope (CytoTrack®, Lyngby, Denmark) within 1 week. The glass disk was mounted in the mounting arm with a spring-lock mechanism. Focus plan was obtained in the DAPI channel at several places on the disk. Scanning was performed with 488 nm Argon-Neon laser, in a spiral pattern with a bandwidth of 10 μm, a process taking ∼2 min. All signals from the Alexa Fluor 488 emission channel were recorded and positive events listed in a hotspot table. Recorded events were visually inspected by the operator in the Alexa Fluor 488 channel and an image gallery was automatically generated using the DAPI, Alexa Fluor 488 and Cy5 channels from positions on the glass slide with possible CTC. The photo gallery was analyzed using the following morphologic criteria: Nearly round and size >4 μm, with visible nucleus within the cytoplasm, DAPI-positive, pan-cytokeratin-positive, CD45-negative. The definition of CTC in the current study is similar to the definition used by other methods analyzing for CTC (12,15–17). Detected cells with the above criteria were termed CTC. All glass plates were scanned and cells meeting the criteria for CTC defined above were counted. Scanning and counting were performed in a blinded setup (Fig. 2B).

### Blood spiking experiments

MCF-7 cells were triturated through an 18-G syringe needle and filtered through a 30-μm nylon mesh (Miltenyi Biotec Ltd., Surrey, UK) to obtain a suspension with a large proportion of single cells. The cells were sorted by a FACS Aria flow cytometer (BD Biosciences) into blood samples from a healthy donor. The number of cells spiked in each blood sample was exactly determined by flow sorting and performed with 10, 33 or 100 cells with nine samples per spike level. In addition, nine control samples where no cells were added were collected. The samples were then processed and analyzed according to the sample preparation and analysis protocol (Fig. 2). It is important for the outcome of a spiking experiment to know the precision and accuracy of the enumeration of added cells to a sample. Therefore, the number of cells actually being sorted directly onto a glass slide by the FACSAria with a setting of 10, 33 or 100 cells was tested with pre-stained cells that were subsequently counted on the fluorescence microscope. The experiment was repeated four times and mean and variance were calculated. The number of cells counted on the glass slides by fluorescence microscopy was considered the actual number of added cells in the spiking experiments and the spiking data were corrected to this value (correction factor). The observed dataset was multiplied with a correction factor. Data were tested by the D'Agostino-Pearson *K*^2^ test Gaussian distribution (18) and was not proven different from a Gaussian distribution. Mean, SD, 95% confidence intervals (95% CI) and percent coefficient of variation (CV%) was calculated by including the variance of the number of cells spiked into a blood sample using Excel.

## Results

**Fig. 3:**
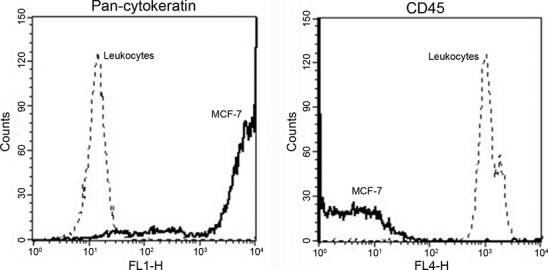
Selective distinction between tumor cells (MCF-7) and leukocytes by staining for cell type-specific markers. Flow cytometer analysis shows specific staining of pan-cytokeratin in epithelial cells (left panel) and CD45 on leukocytes (right panel). The clear discrimination between the two cells types allows us to identify rare circulating tumor cells based on their epithelial marker expression. MCF-7 histograms are shown in bold line, leukocyte histograms in dotted line.

The specificity of the applied antibodies was tested by flow cytometry (Fig. 3). The separation between CTC and leukocytes using the pan-CK marker was excellent, with mean intensities on the scattergram of the two cell types differing almost 1000-fold. This suggests a highly specific staining of CTC and very low background staining for leukocytes. The AE1/AE3 pan-Cytokeratin antibody cocktail used in the current study was chosen for its ability to recognize a broad range of cytokeratins (CK's 1–7, 10, 14, 15, 16 and 19) and has been successfully tested as a marker of CTC (19). The separation between leukocytes and CTC using the CD45 marker was also excellent, with mean intensities on the scattergram of the two cell types differing ∼100-fold.

**Fig. 4:**
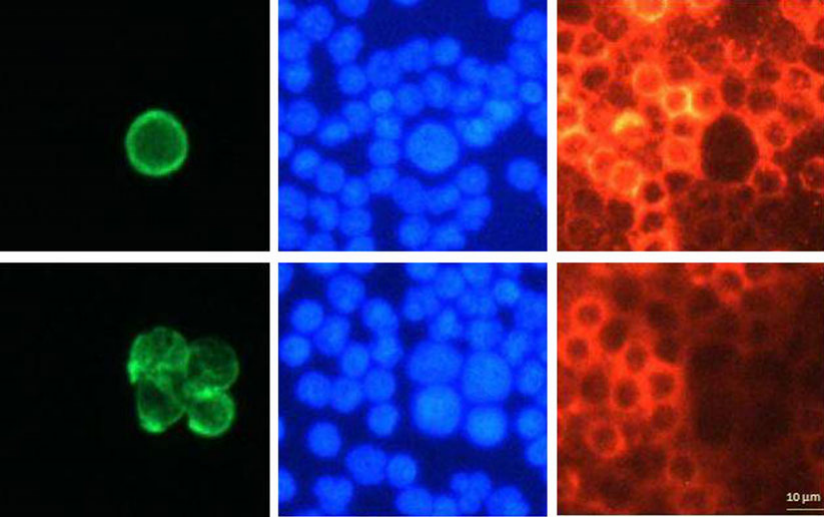
Image galleries of cells detected by the scanning fluorescence microscope from blood spiked with tumor cells (MCF-7 breast cancer cells). The left images show tumor cells stained with a pan-cytokeratin antibody (green), either a single tumor cell (top) or a cluster of four tumor cells (bottom). The center images show corresponding nuclei stained with 4,6-diamidino-2-phenylindole (blue). The left images show corresponding leukocytes stained with a CD45 antibody (red). All images are taken at the same magnification (×40 objective), with scale bar shown at the bottom right.

The reliability and the specificity of antibodies were further tested when cells were analyzed by the CytoTrack® scanning procedure. The scanner uses a glass disk with 60 mm radius as a standard format (Fig. 2). This allows a 15× larger surface for a sample smear compared with standard microscopy slides, and thus potentially allows larger sample volumes for CTC analysis and detection. The scanner has an integrated image acquisition, which provides picture panels where the separation between the DAPI, pan-CK and CD45 stainings are evident (Fig. 4). Antibodies were also tested with isotype controls, which showed very low non-specific binding (data not shown).

**Fig. 5:**
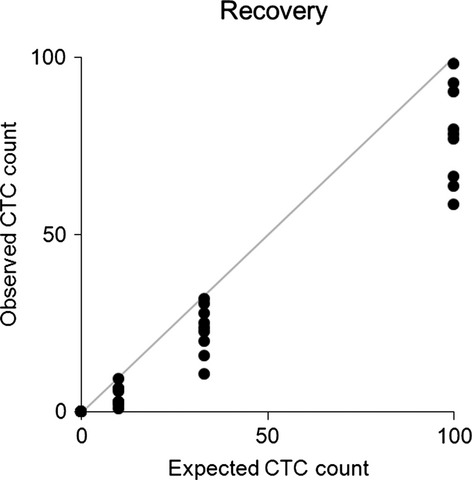
Recovery of CTC (0, 10, 33 and 100 MCF-7 cells) spiked in 7.5 mL whole blood from healthy donor. The number of cells spiked (*x*-axis) is plotted vs the number of cells observed (*y*-axis). Each spike level as well as the controls consists of nine samples. The gray line represents 100% recovery of CTC.

The results of the recovery experiment performed by spiking 10, 33 and 100 tumor cells into three times nine blood samples from a healthy donor showed a recovery rate of 55%, 70% and 78%, respectively. None of the nine control samples analyzed was found to have CTC. The results are shown in Fig. 5. The average percentage of MCF-7 cell recovery was 68% (95% CI = 52–83). The intra-assay coefficient of variation for the recovery rate was between 5% and 72%, and the trend was a lower variation, the more the cells were added. Inter-assay coefficient of variation for the recovery rate for 10, 33 and 100 spiked cells was 59%, 32% and 18%, respectively, with a total coefficient of variation of 36%.

The correlation of the number of observed tumor cells and expected tumor cells was linear and is shown in Fig. 5. Regression analysis yielded a coefficient of determination of *R*^2^ = 0.95 and produced a slope of 0.80.

## Discussion

The current study tested and validated a method for detection and quantification of CTC using a scanning fluorescence microscope (CytoTrack®). The method is different from other methods by the capacity to scan all nucleated cells in a blood sample without using enrichment in a simple procedure with minimal loss of cells. The ability to analyze all cells from a sample without enrichment procedures has prompted the current method validation, as it is expected that simplifying procedures during sample preparation, i.e. avoiding enrichment procedures, could provide good CTC recovery and detection.

Analysis on the scanner gives rise to points of interest (hotspots, Fig. 2), which are visually inspected by the operator in the pan-CK channel and hotspots suspected as CTC selected for automatic image acquisition in the nuclear (DAPI), pan-CK (Alexa488) and CD45 (Cy5) channels. An automatic acquisition of a picture panel from each selected hotspot is generated (Fig. 4). The excellent separation between CTC and leukocytes can be seen in the picture panels.

The recovery of 100 cells spiked in whole blood was 78% (Table 1), which is comparable to other methods, for example, the Cellsearch® method, which find 80–82% recovery (16). The CV% of the 100 spiked cells was 18% (Table 1). This performance of the method using current manual sample preparation is excellent and suggests the scanning fluorescence microscopy method for clinical testing. In comparison, the CV% of Cellsearch® for ∼50 cells is 14–43% (16).

**Table 1:** Recovery and CV% of CTC (10, 33 and 100 MCF-7 cells) spiked in 7.5 mL whole blood from healthy donor

Expected CTC count	Observed CTC count			% recovery			
		
	Average	SD	95% CI	Average	SD	95% CI	CV%
10	5	3	3–8	55	29	36–74	59
33	23	7	18–28	70	21	56–83	32
100	78	14	69–87	78	14	69–87	18
Total	–	–	–	68	23	52–83	36

It is difficult to compare recovery experiments that are not performed in parallel as the spiking methodology can influence the recovery data strongly, as can be exemplified by many studies reporting CTC recovery above 100%, especially in the spiking experiments with low number of cells (20,21).

The recovery of 33 cells spiked in whole blood was 70%, again an excellent recovery with a CV% of 32% (Table 1), reflecting an increased variation of the recovery in the medium number of spiked cells.

The recovery of 10 cells was reduced to 55%, with a CV% of 59% (Table 1). The assay performance on low number of cells should be optimized, as patients with low number of CTC could be missed (false-negatives) by an assay with too high CV%/low recovery. The variation in recovered cell numbers can partially be explained by the difficulty to accurately and reproducibly spike low numbers of cells. Although the buffy coat cells were carefully collected, the loss of cells due to this step cannot entirely be excluded. Also, the lysing of RBC has been shown to reduce WBC with up to 10% (22) and therefore one could expect a reduction in number of CTC to a similar extent.

The most tested and best validated method for detection and isolation of CTCs is the Cellsearch® automated method. The methodology tested in the current paper is comparable with regard to CTC recovery of spiked samples (16). Moreover, the open design of the scanning fluorescence microscope enables the use of custom-made antibody cocktails for each specific application. The technology should be explored further to identify application specific protocols, e.g. for detection of CTC in breast cancer, prostate cancer, ovarian cancer, gastric cancer, colon cancer, etc. CTC analysis is currently much dependent on cytokeratin staining. Therefore, tumor cells with reduced cytokeratin expression will be difficult or impossible to detect using this marker. Moreover, cells double positive for both pan-cytokeratin and CD45 have been reported to have a strong prognostic value for survival (23). Hopefully, it is possible to use other markers, e.g. N-cadherin or Vimentin, to detect the EMT cells (24). The open platform design of the CytoTrack® scanning fluorescence microscope ensures the possibility for researchers to test novel CTC or EMT markers.

In conclusion, the scanning fluorescence microscope is tested and validated with a sample preparation consisting of manual isolation of buffy coat, lysis and subsequent staining. In spite of the use of manual procedures, the method showed excellent recovery and good reproducibility. Thus, the methodology holds great promise as a flexible research tool for finding rare cells in blood. The method should be tested on clinical samples. Optimization and increased reproducibility could be obtained by automation and further simplification of the sample preparation.
